# Circular RNAs and Their Role in Exosomes

**DOI:** 10.3389/fonc.2022.848341

**Published:** 2022-04-28

**Authors:** Zeping Han, Huafang Chen, Zhonghui Guo, Jian Shen, Wenfeng Luo, Fangmei Xie, Yu Wan, Shengbo Wang, Jianhao Li, Jinhua He

**Affiliations:** ^1^Central Laboratory, Guangzhou Panyu Central Hospital, Guangzhou, China; ^2^Department of Laboratory Medicine, Guangzhou Panyu Central Hospital, Guangzhou, China; ^3^Department of Laboratory Medicine, Leizhou Center for Disease Control and Prevention, Leizhou, China; ^4^Department of Gastroenterology, Guangzhou Panyu Central Hospital, Guangzhou, China; ^5^Department of Cardiology, Central Hospital of Panyu District, Guangzhou, China

**Keywords:** non-coding RNA (ncRNA), circRNA, exosome, exosomal circRNAs, disease

## Abstract

As a novel class of endogenous non-coding RNAs discovered in recent years, circular RNAs (circRNAs) are highly conserved and stable covalently closed ring structures with no 5′-end cap or 3′-end poly(A) tail. CircRNAs are formed by reverse splicing, mainly by means of a noose structure or intron complementary pairing. Exosomes are tiny discoid vesicles with a diameter of 40-100 nm that are secreted by cells under physiological and pathological conditions. Exosomes play an important role in cell-cell communication by carrying DNA, microRNAs, mRNAs, proteins and circRNAs. In this review, we summarize the biological functions of circRNAs and exosomes, and further reveal the potential roles of exosomal circRNAs in different diseases, providing a scientific basis for the diagnosis, treatment, and prognosis of a wide variety of diseases.

## Introduction

Non-coding RNAs (ncRNAs) are a class of endogenous RNAs that do not encode proteins, including microRNAs (miRNAs), long non-coding RNAs (lncRNAs), circular RNAs (circRNAs), which are involved in post-transcriptional regulation (1). miRNAs are 18 -22 nucleotides small ncRNAs that modulate the translation of more than 60% of all protein-coding mRNAs in the cell, providing an intermediate regulatory step between gene transcription and translation (2). In contrast with miRNAs, lncRNA segments are longer in sequences, typically more than 200 nucleotides. LncRNAs have similar functions to miRNAs and can regulate gene expressions, such as protein translation and post-transcriptional silencing. Moreover, lncRNAs inhibit the translation of cis and trans genes through histone modifications or disrupting the miRNA regulation (3). CircRNAs were originally considered to be by-products of aberrant splicing or intermediate products of intron lariats that have escaped degradation (4). With the rapid development of various accurate detection technologies, it was found that circRNAs are a kind of ncRNAs molecule composed of hundreds or even thousands of nucleotides, which can directly connect to the 5′- and 3′-ends of linear RNAs, as an intermediate product of the RNA processing reaction. CircRNAs can also be produced by “reverse splicing.” The downstream 5′ splice site (splice donor) is connected to the upstream 3′ splice site (splice receptor), which are found in prokaryotes, eukaryotes, and viruses (5). According to their sequence, circRNAs are divided into three categories: (1) intronic circRNAs (ciRNAs) are composed only of introns and are located mainly in the nucleus (6); (2) exonic circRNAs are produced by the Epstein–Barr virus from the BART gene region and are distributed in the cytoplasm and nucleus (7); and (3) exon–intron circRNAs (EIciRNAs) retain introns between the exons, are located mainly in the nucleus, like circRNAs, and can enhance the expression of parent genes in *cis* (8). Recently, a special type of circRNAs has been identified, called tRNA intronic circular RNAs, which is spliced from endogenous tRNAs under the action of RtcB ligase and the tRNA splicing endonuclease complex (9). CircRNAs have various biological functions and play vital roles in the occurrence, development, invasion, diagnosis, and prognosis of tumors (10–14), and are also important contributors to the development of cardiovascular (15), neurological (16), orthopedic (17), and other diseases.

Exosomes are one of the three main subtypes of extracellular vesicles (EVs) (18), which have attracted considerable attention in clinical research. They are nanoscale round or oval capsule vesicles that are released from cells after the contact and fusion of multiple vesicles with the cell membrane. They were first discovered by Wolf (19) in 1967, who observed vesicle structures he called “platelet dust” that were secreted by cells *in vitro*, with a size of 40–100 nm. In 1983, Pan and Johnstone identified membranous vesicles in the culture medium of reticulocytes and named them exosomes (20). Since then, exosomes have been shown to carry nucleic acids, proteins, lipids, and other biological molecules as important mediators of intercellular communication and its regulation. Their role in the occurrence, development, metastasis, invasion, and drug resistance of tumors has been studied in depth (21), and the content and functions of exosomes are constantly being supplemented and revised.

## Function of circRNAs

### MiRNA Sponges

CircRNAs are competitive endogenous RNA molecules with abundant miRNA binding sites, which are also called miRNA response elements, that can competitively bind to miRNAs to remove their inhibitory effects on downstream target genes, thereby affecting intracellular signal transduction pathways and the expression of target genes (22). The role of circRNAs as miRNA sponges is the classic model of their function. For example, Hansen et al. identified the circular RNA sponge for miRNA (miR)-7 (ciRS-7) (23), also known as miRNA sponge cerebellar degeneration-related protein 1 antisense, which negatively regulates miR-7 expression. In addition, more than 70 miR-7 binding sites have been found on ciRS-7 (24), which is highly expressed in HEK293 cells and can bind up to 20,000 miR-7 molecules in each cell. Other studies have confirmed that the downregulation of circRNA-PVT1 inhibits the expression of sirtuin 7 by upregulating the expression of miR-3666, and finally inhibits the proliferation and metastasis of hepatocellular carcinoma cells (25). Moreover, circRNAs not only act as miRNA sponges in humans but also in parasites such as nematodes (26). Some circRNAs with miRNA sponge function have been found in plants such as *Arabidopsis* (27), wheat (28), and citrus (29).

### Regulation of Gene Transcription

CiRNAs and EIciRNAs competitively regulate the transcription of parental genes through linear splicing. CiRNAs can act as active regulators of RNA polymerase (Pol) II to regulate the transcription of parental genes (30). EIciRNAs can interact with U1 small nuclear RNA (snRNP) to form the EIciRNA-U1 snRNP complex, which then interacts with the transcriptional complex of RNA Pol II to affect parental gene expression (8, 31). In addition, non-coding intron transcripts, such as ci-ankrd52, act as positive regulators of RNA Pol II transcription, have *cis*-regulatory effects on their parental coding genes, and are associated with a mechanism for the extension of RNA Pol II (6). Subsequently, a study in 2020 showed that circ-DAB1 upregulates the expression of recombination signal-binding protein for immunoglobulin kappa J region (RBPJ), which leads to the increased binding of RBPJ to the DAB adaptor protein 1 (DAB1) promoter, thereby activating the transcription of the parental gene, DAB1 (32).

### Interaction With Proteins

CircRNAs can also act as sponges of proteins and combine with RNA-binding proteins (RBPs) to form RNA-protein complexes that affect protein expression (33). For example, the RNA splicing factor MBL is an RBP that binds to the second exon of its parent gene and promotes its cyclization to form circ-MBL in drosophila (31). At the same time, there are multiple sites on circ-MBL that bind to MBL protein, which reduces the effective concentration of MBL (31). CircRNAs can also interact with specific target proteins and participate in cell proliferation, differentiation, and apoptosis (34). For example, circ-Foxo3, cyclin-dependent kinase inhibitor 1 (p21), and cyclin-dependent kinase 2 (CDK2) form the ternary complex circ-Foxo3-p21-CDK2, which inhibits the function of CDK2 and blocks the cell cycle. In addition, directly silencing endogenous circ-Foxo3 promotes cell proliferation (35). Another report shows that the circ-ANRIL can bind to PES1 protein, thereby preventing pre-rRNA binding and exonuclease-mediated rRNA maturation. Consequently, circANRIL impairs ribosome biogenesis, leading to activation of p53 and a subsequent increase in apoptosis and decrease in proliferative rate (36). Because proteins have many functions, the other effects of circRNAs binding to proteins need to be studied in more depth.

### Participation in Translation

**Figure 1:**
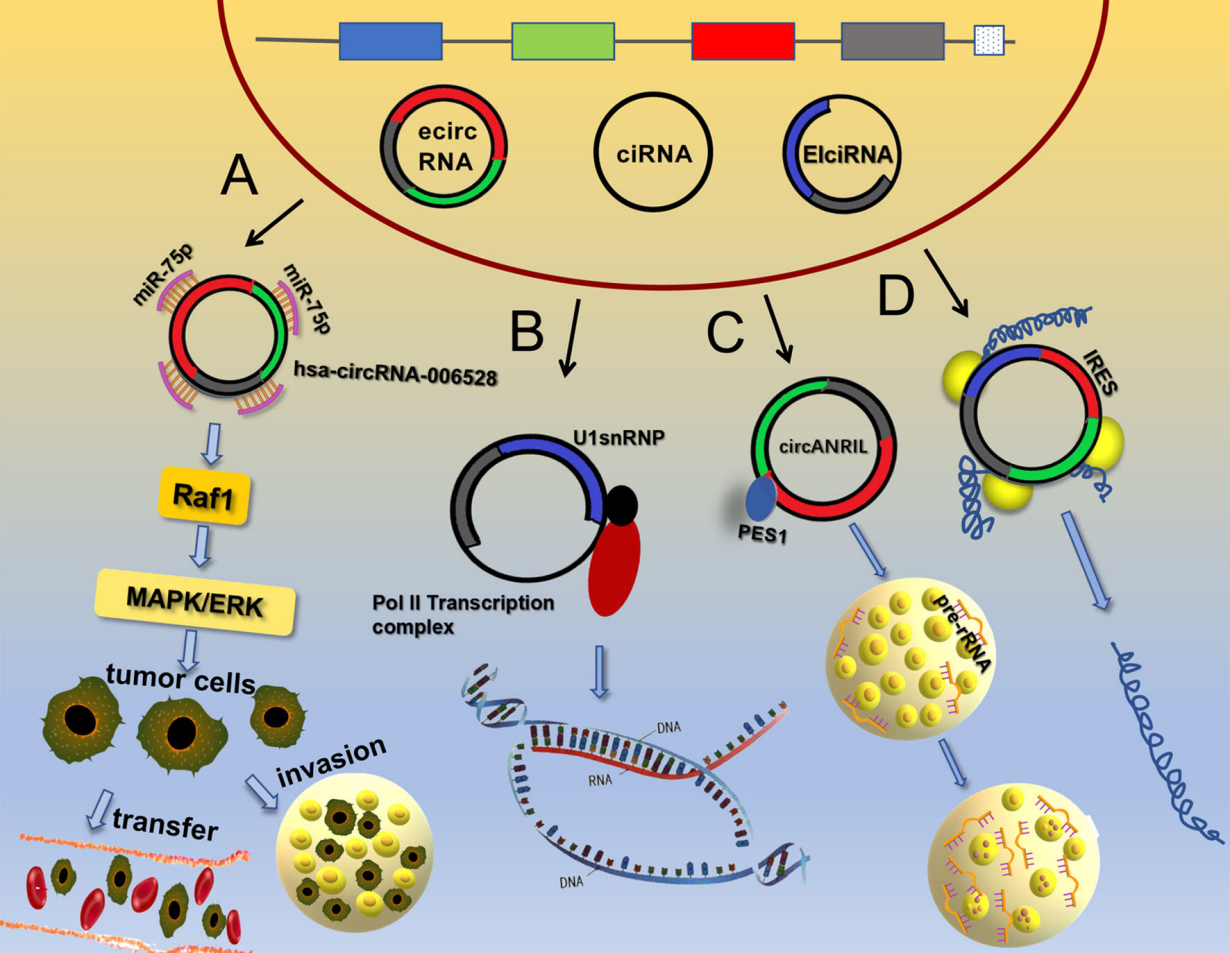
Main functions of circRNAs. **(A)** CircRNAs can adsorb miRNAs to activate signaling pathways. **(B)** EIciRNA and U1 snRNP form a complex that interacts with the RNA Pol II complex to regulate gene transcription. **(C)** Circ-ANRIL binds to PES1 protein to increase the number of cell nucleoli and pre-rRNA accumulation, thereby promoting apoptosis. **(D)** CircRNAs can be translated into protein through an IRES.

Initially, circRNAs were considered as non-coding RNA molecules, but in recent years, many studies have shown that circRNAs have roles in protein translation (37–40), which disproved a long-believed concept. It was originally thought that eukaryotic ribosomes could initiate translation from circRNAs only if the circRNAs contained an internal ribosome entry site (IRES) (41). For example, circ-PINT can be translated *via* its IRES into the PINT87aa polypeptide, which contains 87 amino acids. PINT87aa binds to the polymerase-associated factor complex gene and inhibits the development of malignant glioma (42). The IRES domain of circRNAs was subsequently predicted to be a binding site for many RBPs, including HUR and PTB, which modulate the translation of IRES element-driven proteins (43). As research continues to advance, it has been discovered that circRNAs without IRES components can be translated into multiple functional proteins. For example, N6-methyladenosine (m^6^A) not only affects mRNA translation under heat shock stress conditions (44) but also regulates the translation of circRNAs. The presumed mechanism is m^6^A modification by the METTL3/METTL14-WTAP protein complex, the regulation of m^6^A de-modification by FTO, and the site containing the m^6^A modification ultimately initiates protein translation by recruiting YTHDF3 and thus eIF4G2 (45), which fills a gap in the study of the chemical modifications of circRNAs. In addition, it was discovered that circRNAs can use overlapping codons to translate proteins, which is a unique method of translation (46). For example, circ-AKT3 uses overlapping codons to generate a new functional protein, AKT3-174aa. This protein affects the phosphorylation of AKT2/3 molecules by binding to p-PDK1, and then negatively regulates the PI3K/AKT signaling pathway, inhibiting the occurrence and development of brain tumors (47). This unique translation process has significance for the study of new functions of circRNAs (**Figure 1**).

## CircRNAs and Exosomes

### Mechanism by Which circRNAs Enter Exosomes

In 2015, it was first reported that circRNAs are enriched in exosomes and have good stability (48). The association of circRNAs with exosomes, followed by genome-wide analysis of RNA-sequencing data, showed that the abundance of circRNAs in exosomes and the circular–linear splicing rate is increased by at least 2- to 6-fold compared with their levels in producer cells, suggesting that circRNAs are actively incorporated into exosomes. Furthermore, there are more than 1,000 different circRNA candidates in human serum exosomes (49). Dou et al. used three colon cancer cell lines to show that circRNAs are more abundant in exosomes than in cells. In addition, different mutations of KRAS (proto-oncogene) were shown to have different effects on the exosomal content of circRNAs. These findings indicate that a large number of circRNAs are present in exosomes and that KRAS mutations affect their abundance (50).

**Figure 2:**
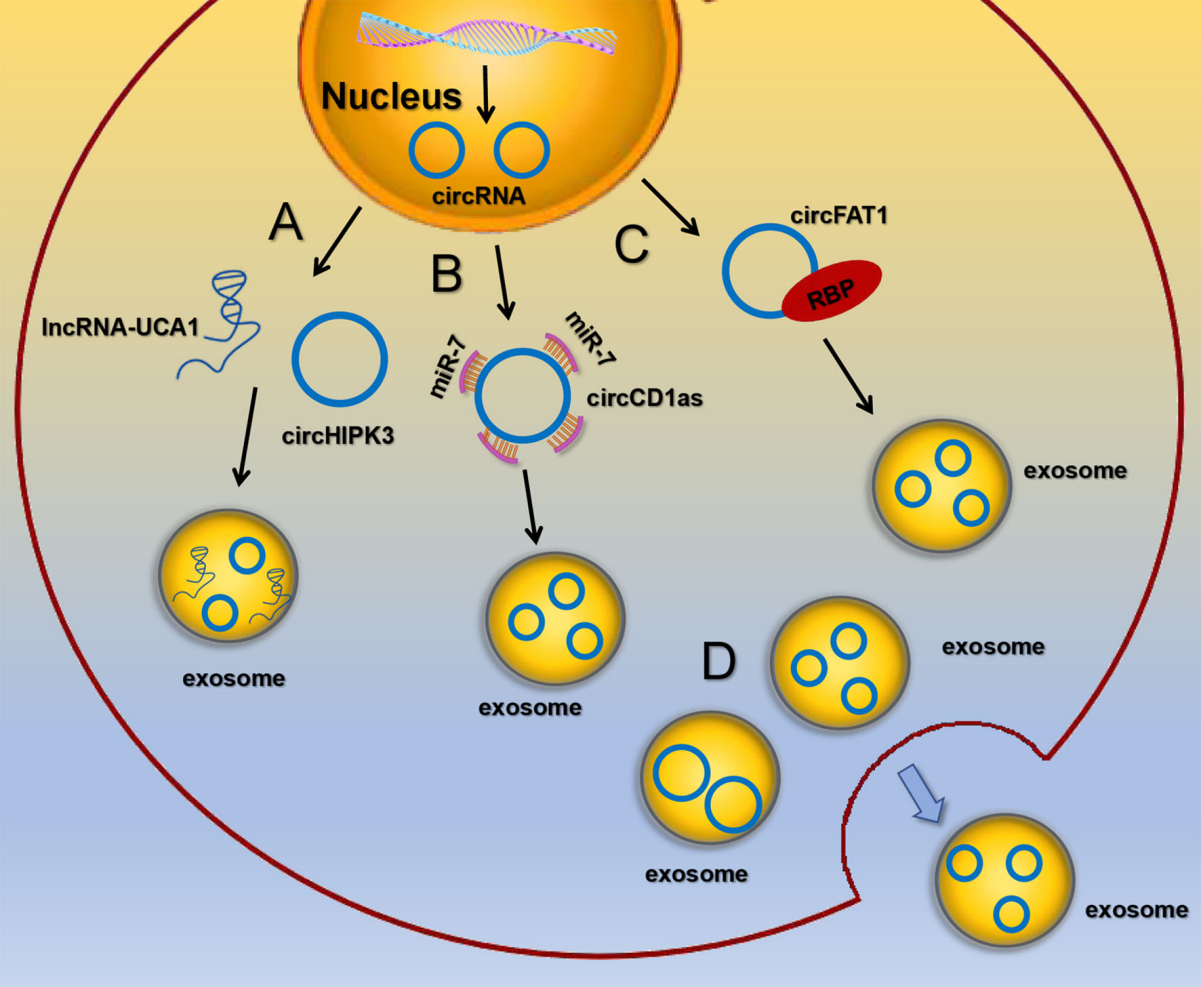
The sorting and secretion mechanisms of circRNA in exosomes **(A)** lncRNAs competitively regulate circRNAs sorting into exosomes. **(B)** circRNAs act as miRNA sponges. **(C)** RBPs recognize RNA with specific binding sequences and regulate the sorting of exosomal circRNAs. **(D)** Exosomes preferentially release smaller circRNAs.

Although the presence of circRNAs in exosomes has been confirmed, the mechanism by which circRNAs enter exosomes remains unclear. It has been found that circRNAs are selectively packaged into EVs, such as exosomes and microvesicles, but it is more pronounced in exosomes. circRNAs sorting into exosomes may be regulated by the following mechanisms: (1) lncRNAs competitively regulate circRNAs sorting into exosomes. Barbagallo C et al. found that knockdown of lncRNA UCA1 in serum exosomes could inhibit mitogen activated protein kinase (MAPK) signaling pathway, resulting in up regulation of circHIPK3 expression, suggesting that the competitive mechanism of lncRNA UCA1 may regulate the sorting of circHIPK3 (51). (2) circRNAs act as miRNA sponges. It’s reported that exosomal circCDR1as acted as a sponge for miR-7. When miR-7 was ectopically expressed in liver cancer cells, the expression level of circCDR1as in exosomes was significantly down-regulated, while the expression of circCDR1as in cells was increased (48). (3) RBPs recognize RNA with specific binding sequences and regulate the sorting of exosomal circRNAs. DKs-8 cells secreted exosomes enrich RBPs, which were involved in regulating the sorting process of circRNAs by binding with circFAT1 (50). (4) Exosomes preferentially release smaller circRNAs. Cells secrete exosomes circRNAs and release them to the extracellular environment may be related to the size of circRNAs. Preußer C et al. found that the average size of circRNAs that were not secreted from cells was 459 nts (52), while the average size of circRNAs released by exosomes was 435 nts, suggesting that size appears to be an important determinant for selective vesicle export of circRNAs (**Figure 2**). Nevertheless, the exact mechanism regarding the sorting and release of circRNAs is still largely unknown and awaits further study.

### Clearance Mechanism or Information Exchange Channel?

CircRNAs are highly stable, evolutionarily conserved, and have no 3′- and 5′-ends (53). They also have built-in resistance to the major enzymes responsible for mRNA degradation, and thus may accumulate in cells. Lasda and Parker (54) analyzed the relative number of circRNAs and linear RNAs in EVs (including exosomes and microvesicles) in HeLa, 239T, and U-2OS cell lines. They found that circRNAs were more abundant compared with linear RNAs. A large number of circRNAs can be packaged into EVs, which bind to EVs and co-precipitate for their removal from cells. In addition, reducing the accumulation of circRNAs induced by reverse splicing may allow them to be exported preferentially from cells. This may be a mechanism for clearing accumulated circRNAs from cells. Furthermore, the authors suspected that there are other mechanisms for the removal or degradation of circRNAs, including transport mechanisms of other vesicles or endonuclease cleavage. Recently, a new process for RNA degradation mediated by m^6^A has been identified that can be applied to mRNAs and circRNAs. The m^6^A-recognition protein YTHDF2 binds to target molecules and recruits HRSP12. CircRNAs can be bound to and be degraded through the YTHDF2-HRSP12-mediated RNase P/MRP complex (55).

Exosomes or microvesicles can be taken up by other cells. They stimulate target cells directly through receptor-mediated interactions, and also transfer biologically active molecules such as membrane receptors, proteins, and mRNAs from source cells to target cells. Exosomes or microvesicles participate in communication between cells under physiological and pathological conditions (56). Therefore, Lasda and Parker believed that, in some cases, circRNAs may be packaged into EVs for cell-to-cell communication. Another study also suggested that the mechanism for the selective release of circRNAs is a way to transfer information from donor cells to recipient cells (52). However, the elimination mechanism and the signal communication pathway need to be explored further and confirmed.

### Study of Exosomal circRNAs in Tumors

Cancer is a global public health problem that poses a serious threat to human life and physical health, resulting in significant social and economic burdens. According to a report by GLOBOCAN, in 2020, the number of new cancer cases worldwide reached 19.3 million, and approximately 10 million people died from cancer. In addition, breast cancer has replaced lung cancer as the most common cancer worldwide, and it is estimated that by 2040, the global cancer burden will be 28.4 million cases, an increase of 47% compared to 2020 (57). Exosomal circRNAs play an important role in a wide range of pathological processes, especially in a variety of tumors, including occurrence, development, invasion, and other processes, and might make a significant contribution to the diagnosis and prognosis of tumors (58). **Table 1** summarizes some of the functions and clinical significance of exosomal circRNAs in a range of different tumors.

**Table 1:** Function and clinical significance of exosomal circRNAs in tumors.

Function	Exosomal circRNA	Tumor type	Expression	Mechanisms	Functional or clinical applications	Reference
Regulate cell proliferation	circ-MEMO1	NSCLC	Upregulated	MiR-101-3p/KRAS	Accelerate proliferation, cell cycle progression, and glycolytic metabolism and inhibit apoptosis	(59)
	circRASSF2	LSCC	Upregulated	miR-302b-3p/IGF-1R	Promote proliferation	(60)
	circ-MMP1	Glioma	Upregulated	miR-433/HMGB3	Promote the proliferation and movement of glioma cells and inhibits apoptosis	(61)
	circ-DB	HCC	Upregulated	miR-34a/USP 7/CyclinA 2	Promote cell growth and inhibits DNA damage	(62)
	circ-FBLIM1	HCC	Upregulated	miR-338/LRP6	Promote the progression and glycolysis	(63)
	circ-0051443	HCC	Downregulated	MiR-331/BAK1	Promote cell apoptosis and arrest the cell cycle	(64)
	circ_0000199	OSCC	Upregulated	miR-145-5p/miR-29b-3p	Promote proliferation and inhibits apoptosis	(65)
	circ_400068	RCC	Upregulated	miR-210-5p/SOCS1	Promoted the proliferation and inhibited the apoptosis of healthy kidney cells	(66)
	circ-G042080	MM	Upregulated	hsa-miR-4268/TLR4	Induces autophagic death of cardiomyocytes	(67)
Regulate invasion and metastasis	circIFT80	CRC	Upregulated	miRNA-1236-3p/HOXB7	Promote proliferation, migration and invasion	(68)
	circFMN2	CRC	Upregulated	miR-1182/hTERT	Promote proliferation and migration	(69)
	circ-ABCC1	CRC	Upregulated	β-catenin/Wnt pathway	Promote cell stemness, sphere formation and metastasis	(70)
	circ-PACRGL	CRC	Upregulated	miR-142-3p/miR-506-3p-TGF-β1	Promotes cell proliferation, migration, and invasion	(71)
	circFNDC3B	CRC	Downregulated	VEGFR	Inhibits angiogenesis and cancer progression	(72)
	circFNDC3B	PTC	Upregulated	miR-1178/TLR4	Promote cell proliferation, migration, and invasion	(73)
	ciRS-133	CRC	Upregulated	miR-133a/GEF-H1/RhoA	Promote tumor metastasis	(74)
	circANTXR1	HCC	Upregulated	miR-532-5p/XRCC5	Promote proliferation and metastasis	(75)
	circRNA Cdr1as	HCC	Upregulated	miR-1270/AFP	Enhance proliferation and migration	(76)
	circ-100338	HCC	Upregulated	interact with NOVA2	Promote angiogenesis and metastasis	(77)
	circ-PTGR1	HCC	Upregulated	miR449a/EMT	Promote migration and invasion	(78)
	circ-0072088	HCC	Upregulated	miR-375/MMP-16	Promote invasion and Migration	(79)
	circ-0004277	HCC	Upregulated	inhibit ZO-1	Promote EMT progression	(80)
	circ-IARS	PDAC	Upregulated	miR-122/RhoA/ZO-1	Promote tumor invasion and metastasis	(81)
	circ-PDE8A	PDAC	Upregulated	miR-338/MACC1/MET	Promote proliferation and invasion	(82)
	circ-PRMT5	UCB	Upregulated	miR-30c/SNAIL1/E-cadherin	Promote growth and metastasis	(83)
	circ-NRIP1	GC	Upregulated	miR-149-5p/AKT1/mTOR	Promotes tumor metastasis	(84)
	circ-RanGAP1	GC	Upregulated	miR-877-3p/VEGFA	Promote invasion and metastasis	(85)
	circSHKBP1	GC	Upregulated	miR-582-3p/HUR/VEGF	Promote proliferation, migration, invasion and angiogenesis	(86)
	circSATB2	NSCLC	Upregulated	miR-326/FSCN1	Promotes proliferation, migration and invasion	(87)
	FECR1	SCLC	Upregulated	miR584/ROCK1	Promote tumor metastasis	(88)
	circSETDB1	LUAD	Upregulated	miR-7/Sp1/E-cadherin	Promote proliferation, migration, invasion	(89)
	circPUM1	ovarian cancer	Upregulated	miR-615-5p/miR-6753-5p/NF-Kb/MMP2	Promote proliferation, migration and invasion	(90)
	circWHSC1	ovarian cancer	Upregulated	miR-145/miR-1182	Promote proliferation and metastasis	(91)
	circ_0000284	cholangiocarcinoma	Upregulated	miR-637/LY6E	Enhance migration, invasion and proliferation	(92)
	circ_0044516	PC	Upregulated	miR-29a-3p	Promote proliferation and metastasis	(93)
Regulate treatment resistance	has_circ_0002130	NSCLC	Upregulated	miR-498/GLUT1/HK2/LDHA	Contribute to Osimertinib-resistant	(94)
	hsa_circ_0014235	NSCLC	Upregulated	miR-520a-5p/CDK4	Promotes cisplatin resistance in cells and malignant growth	(95)
	circRNA_102481	NSCLC	Upregulated	miR-30a-5p/ROR1	contribute to EGFR-TKIs resistance	(96)
	circUHRF1	HCC	Upregulated	miR-449c-5p/TIM-3	promote immune escape and PD1 immunotherapy resistance	(97)
	circRNA-SORE	HCC	Upregulated	YBS1	contribute to sorafenib resistance	(98)
	circTMEM181	HCC	Upregulated	miR-488-3p/CD39	Contributes to immunosuppression and anti-PD1 resistance	(99)
	ciRS-122	CRC	Upregulated	miR-122/PKM2	Promote glycolysis and Oxaliplatin resistance	(100)
	hsa_circ_0000338	CRC	Upregulated	–	Increase FOLFOX sensitivity	(101)
	circ-Foxp1	Ovarian cancer	Upregulated	miR-22/miR-150-3p	Promotes cell proliferation and cisplatin resistance	(102)
	circRNA Cdr1as	Ovarian cancer	Downregulated	miR-1270/SCAI	Inhibit proliferation and promote cisplatin-induced cell apoptosis	(103)
	circ-PVT1	GC	Upregulated	miR-30a-5p/YAP1	Enhance drug resistance to Cisplatin	(104)
	circ_0032821	GC	Upregulated	miR-515-5p/SOX9	Enhances resistance to Oxaliplatin	(105)
	circNFIX	Glioma	Upregulated	miR-132/ABCG2	Enhance drug resistance to Temozolomide	(106)
	circ-HIPK3	Glioma	Upregulated	miR-421/ZIC5	Enhance drug resistance to Temozolomide	(107)
	circATP8B4	Glioma	Upregulated	miR−766	Promote cell radioresistance	(108)
	hsa_circ_103801	Osteosarcoma	Upregulated	–	Enhance drug resistance to Cisplatin	(109)
	circ_UBE2D2	Breast Cancer	Upregulated	miR-200a-3p	Improves drug resistance of breast cancer to tamoxifen	(110)

NSCLC, non small cell lung cancer; LSCC, laryngeal squamous cell carcinoma; HCC, hepatocellular carcinoma; OSCC, oral squamous cell carcinoma; RCC, renal cell carcinoma; MM, multiple myeloma; CRC, colorectal cancer; PTC, papillary thyroid carcinoma; PDAC, pancreatic ductal adenocarcinoma; UCB, urothelial carcinoma of the bladder; GC, gastric cancer; SCLC, small cell lung cancer; LUAD, lung adenocarcinoma; PC, prostate cancer.

#### Regulae Cell Proliferation

As dysproliferation is one of the important factors in tumor transformation, the regulation mechanism of the cell cycle has received increasing attention (111). In recent years, an increasing number of exosomal circRNAs have been revealed to regulate cell proliferation in various cancers. Ding C et al. found that the level of circ-MEMO1 in serum of patients with non-small cell lung cancer (NSCLC) was higher than that in healthy people (59). Knockout of circ-MEMO1 inhibited NSCLC cell proliferation and blocked cell cycle in G0/G1 phase, while up-regulated circ-MEMO1 expression promoted cell proliferation, cell cycle progression and glycolysis metabolism and inhibited cell apoptosis. The possible mechanism might be exosomal circ-MEMO1 up-regulated KRAS expression in NSCLC cells by competitively binding miR-101-3p. Another study showed that exosomal circRASSF2 could promote the progression of laryngeal squamous cell carcinoma (LSCC). Compared with the control group, the expression of circRASSF2 in tumor tissues and serum exosomes were significantly up-regulated, and the down-regulation of exosomal circRASSF2 through the miRNA-302b-3p/IGF-1R axis could significantly suppress cell proliferation (60). Yin K et al. found that CircMMP1 was abnormally up-regulated in glioma tissues and serum exosomes compared with corresponding counterparts, and CircMMP1 promoted the proliferation and motility and impeded the apoptosis of glioma cells by enhanced high mobility group box 3 (HMGB3) level through sponging miR-433 (61). Exosomal circDB was up-regulated in hepatocellular carcinoma (HCC) patients with a high body fat rate. Exosomal circDB secreted by adipocytes could regulate deubiquitination of HCC by inhibiting miR-34a and activating USP7/Cyclin A2 signaling pathway, which promoted cell growth and reduced DNA damage (62). Lai Z et al. reported that CircFBLIM1 was highly expressed in HCC serum exosomes and HCC cells (63). Their further findings verified that exosomal circFBLIM1 contributed to the progression and glycolysis of HCC by sponging miR-338 and upregulating LRP6 expression. Chen W et al. show that circ0051443 expression was significantly lower in the plasma exosomes and tissues from patients with HCC than healthy controls (64). Exosomes containing circ-0051443 were secreted from normal cells, with exosomal circ-0051443 transported from normal cells to HCC cells. Exogenous circ-0051443 bound to miR-331-3p competitively and reduced the expression of Bcl2 Antagonist/Killer 1 (BAK1), a crucial cell death regulator, thereby suppressing the malignant biological behaviors by promoting cell apoptosis and arresting the cell cycle.

#### Regulate Invasion and Metastasis

Exosomes can mediate molecular communication and material transfer between primary tumor sites and distant metastasis sites. By regulating a series of cell activities including epithelial mesenchymal transition (EMT) and angiogenesis, exosomes play crucial roles in tumor cell metastasis and invasion (112, 113). In colorectal cancer (CRC), circIFT80 accelerated the tumor progression by entering exosomes promoting CRC cell growth, migration, and invasion through mir-1236-3p/HOXB7 axis (68). circFMN2 was highly expressed in serum exosomes of patients with CRC and negatively correlated with the level of miRNA-1182. circFMN2 could increase the expression of hTERT by binding with miR-1182, which significantly promoted the proliferation and migration of CRC cells, suggesting that exosomal circFMN2 play an important role in promoting the growth of colorectal cancer (69). Exosomes from CD133+ CRC cells carrying circ-ABCC1 could activate the Wnt/β-catenin pathway to mediate cell stemness and metastasis in CRC (70). Shang A et al. also demonstrated that CRC-derived exosomes promote CRC proliferation, migration, and invasion (71). Their further research confirmed that circPACRGL was significantly up-regulated in CRC cells with tumor-derived exosomes addition. And CRC-derived exosomal circPACRGL regulated differentiation of N1-N2 neutrophils and promoted CRC proliferation, migration, and invasion *via* miR-142-3p/miR-506-3p-TGF-β1 axis. Zeng W et al. found that the expression of circFNDC3B was significantly decreased in CRC tissues, CRC cell lines and exosomes. circFNDC3B-enriched exosomes inhibited angiogenesis and CRC progression (72). This study also demonstrated that circFNDC3B-enriched exosomes could repress angiogenesis and CRC progression by decreasing VEGFR expression. Interestingly, another research showed that circFNDC3B was highly expressed in extracted serum exosomes derived from papillary thyroid cancer (PTC) patients, and the expression levels of circFNDC3B and Mir-1178 have significantly negatively correlated (73). Their further research demonstrated that circFNDC3B inhibited PTC cell proliferation, migration, and invasion and promoted cell apoptosis through the miR-1178/TLR4 pathway. In addition to making cancer cells more invasive, exosomal circRNAs can also promote the invasion of cancer cells by changing the tumor microenvironment. Huang C et al. found that exosome-mediated the intercellular transmission of circANTXR1 in HCC cells (75). Overexpressed exosomal circANTXR1 could depress miR-532-5p expression and promote XRCC5 mRNA and protein expression, promoting the proliferation, migration and invasion of HCCLM3 cells. Another research suggested that exosomal circRNA Cdr1as from HCC cells enhanced circRNA Cdr1as expression and accelerated proliferative and migratory abilities to surround normal cells (76). It’s reported that exosomal circRNA-100338 was up-regulated in a highly metastatic HCC cell line. The metastatic ability of HCC cells could be enhanced by transferring exosomal circRNA-100338 to recipient human umbilical vein endothelial cells (HUVECs) and also promoted cell proliferation, angiogenesis, permeability, and vasculogenic mimicry (VM) formation ability and tumor metastasis (77).

#### Regulate Treatment Resistance

Nowadays, the combination of radiotherapy and chemotherapy after surgical resection is the main clinical treatment of malignant tumors. Although the initial efficacy is remarkable, with the emergence of chemoresistance, it has become a huge obstacle to the prognosis. Studies have confirmed that epigenetic variation, oncogene activation, anti-oncogene inactivation, tumor heterogeneity and apoptosis dysregulation are the main causes of tumor drug resistance (114). Current studies have shown that circRNAs can play crucial roles in mediating tumor chemoresistance (115), and exosomes, as essential mediators of communication between tumor cells or between tumor cells and stromal cells, can play a role in the transmission of chemoresistance through the transfer of circRNA (86). At present, the study of circRNA-mediated tumor treatment resistance through exosome delivery is at the forefront of academic research and has important scientific significance.

In serum exosomes of osimertinib-resistance NSCLC patients, Ma J et al. found that the expression of hsa_circ_0002130 was up-regulated, while knockdown of hsa_circ_0002130 significantly inhibited cell proliferation, glycolysis, and promoted cell apoptosis in osimertinib-resistant NSCLC, thereby inhibiting tumor growth (94). The mechanism was to regulate the expression of glucose transporter 1 (GLUT1), hexokinase-2 (HK2) and lactate dehydrogenase A (LDHA) by sponging miR-498, which promoted osimertinib-resistant NSCLC cells to respond to osimertinib-resistance. Hence, hsa_circ_0002130 could act as a new therapeutic target for osimertinib-resistant NSCLC. HCC-derived exosomal circUHRF1 expression was elevated compared with healthy control and was associated with poor clinical prognosis and natural killer (NK) cells dysfunction. Additionally, exosomal circUHRF1 could inhibit IFN-γ and TNF-α secretion in NK cells by upregulating the expression of T cell immunoglobulin and mucin domain 3 (TIM-3) *via* degradation of miR-449c-5p, thereby promoting HCC immune avoidance and anti-PD1 immunotherapy resistance (97). Wang X et al. demonstrated that ciRS-122 could be transferred from oxaliplatin-resistant CRC cell exosomes to sensitive cells, which increased the expression of the M2 isoform of pyruvate kinase (PKM2) by suppressing miR-122, then promoting glycolysis and drug resistance (100). In addition, si-ciRS-122 could block the ciRS-122/miR-122/PKM2 axis at the post-transcriptional level and reverse the resistance of CRC cells to oxaliplatin. In epithelial ovarian cancer (EOC) patients, the expression of exosomal circFoxp1 in cisplatin (DDP)-resistant patients was significantly higher than that in DDP-sensitive patients. Overexpressed exosomal circFoxp1 could enhance the survival and proliferation of EOC cells and promote the resistance of EOC cells to DDP, which could be an independent predictor of survival outcome and DDP resistance in EOC patients. The mechanism might be that the expression of CCAAT enhancer binding protein gamma (CEBPG) and formin like 3 (FMNL3) are positively regulated by miR-22 and miR-150-3p (102). Furthermore, Zhao M et al. found that circATP8B4 from radioresistant U251 extracellular vesicles might be transferred to normal glioma U251 cells and act as an miR−766 sponge to promote cell radioresistance (108). The above studies suggest that a variety of exosomal circRNAs are closely related to treatment resistance and may become a new target for tumor therapy.

### Effects of Exosomal circRNAs in Other Diseases

Exosomal circRNAs not only play a role in a variety of biological processes in tumors but also have an impact on the mechanisms of a variety of other diseases (116). For instance, 5,095 circRNAs were identified in cerebrospinal fluid-derived exosomes from patients with immune-mediated demyelinating disease. Among them, the expression of 26 circRNAs was found to be significantly different in cerebrospinal fluid exosomes. Further study showed that hsa_circ_0087862 and hsa_circ_0012077 could act as molecular markers for the diagnosis of immune-mediated demyelinating disease and could also indicate cellular metabolism and disease progression (117). Secondly, circrNA-EP400, significantly increased in M2 macrophage exosomes, was reported to inhibit miR-15b-5p expression and increase the expression of fibroblast growth factor (FGF)-1/7/9, thereby promoting fibrosis, proliferation, and migration of fibroblasts and tenocytes (118). This study provides a new therapy for tendon injury. In polycystic ovary syndrome, the expression level of exosomal circLDLR decreased significantly. As an important mediator, circLDLR could directly bind to miR-129, inhibit the expression of CYP19A1 in KGN cells, and reduce the secretion of estradiol (119). Exosomal circ-Ehmt1 regulates retinal microvascular dysfunction through the NFIA/NLRP3 signaling pathway by inhibiting the formation of NLRP3 inflammasomes in endothelial cells to protect endothelial cells from high glucose-induced damage, which could be a therapeutic target for diabetic retinopathy (120). Besides, some differentially expressed exosomal circRNAs with miRNA-binding sites in umbilical cord blood are essential for the progression of gestational diabetes and fetal growth and development (121). Exosomal circ_0000253 is upregulated in nucleus pulposus cells (NPCs) during intervertebral disc degeneration, and competitively adsorbs miRNA-141-5p and downregulates SIRT1 expression to promote the apoptosis of NPCs and inhibit the proliferation, blocking circRNA_0000253 as a potential treatment for IDD (122). Notably, exosomal circRNAs have also been demonstrated to have a role in the pathogenesis of Corona Virus Disease 2019 (COVID-19). Studies have shown that differentially expressed circRNAs and long non-coding RNAs in exosomes may be involved in regulating host cell immunity and inflammation, substance and energy metabolism, cell cycle, and apoptosis (123). All of these studies provide a new direction for the diagnosis and treatment of various diseases. **Table 2** summarizes some of the functions and clinical significance of exosomal circRNAs in other diseases.

**Table 2:** Function and clinical significance of exosomal circRNAs in other diseases.

Exosomal circRNA	Disease	Expression	Mechanisms	Functional or clinical applications	Reference
circRNA-Ep400	TI	Upregulated	miR-15b-5p/FGF-1/7/9	Promoted fibrosis, proliferation, and migration of fibroblasts and tenocytes	(118)
circLDLR	PCOS	Downregulated	miR-129/CYP19A1	Reduce the secretion of estradiol	(119)
circ-Ehmt1	DR	Upregulated	NFIA/NLRP3	Reduced HG-induced endotheliocyte injury	(120)
circ_0000253	IDD	Upregulated	miRNA-141-5p/SIRT1	Promote apoptosis of NPCs and inhibit proliferation	(122)
circEDIL3	RA	Upregulated	miR-485-3p/PIAS3/STAT3/VEGF	Suppress inflammation-induced angiogenesis and promotes pannus progression	(124)
hsa_circRNA_104484	Sepsis	Upregulated	hsa-miR-378a-3p/hsa-miR-378d	Participate in the processes of inflammation and immune regulation	(125)
circRNA_0002113	MI	Upregulated	miR-188-3p/RUNX1	Mediate cell apoptosis, suppress myocardial infarction	(126)
circ-Rtn4	Osteoporosis	Upregulated	miR-146a	Attenuate TNF-α-induced cytotoxicity and apoptosis	(127)
circ-BRWD1	OA	Upregulated	miR-1277/TRAF6	Impede chondrocyte viability and facilitate apoptosis, inflammation and ECM degradation	(128)
circRNA_0001236	OA	Upregulated	miR-3677-3p/Sox9	Enhance cartilage repair and suppress cartilage degradation	(129)
circRNA-0006896	AS	Upregulated	miR-1264/DNMT1/SOCS3/JNK/STAT3	Promote the proliferation and migration of HUVECs, influence vulnerable plaque formation	(130)
circTAOK1	DN	Upregulated	miR-520h/SMAD3	Boost proliferation, fibrosis, and EMT of GMC	(131)
circ_DLGAP4	DKD	Upregulated	miR-143/ERBB3/NF-κB/MMP-2	Promote proliferation and fibrosis of MCs	(132)
mmu_circ_0000623	Liver fibrosis	Downregulated	miR-125/ATG4D	Activate autophagy and suppress hepatic fibrosis	(133)
circ-ZC3H6	pSS	Upregulated	hsa-miR-142-3p	As noninvasive biomarkers for the diagnosis of pSS	(134)

TI, tendon injury; PCOS, polycystic ovary syndrome; DR, Diabetic retinopathy; IDD, intervertebral disc degeneration; RA, rheumatoid arthritis; MI, myocardial infarction; OA, osteoarthritis; AS, atherosclerosis; DN, diabetic nephropathy; DKD, diabetic kidney disease; PSS, primary Sjogren’s Syndrome.

### Exosomal circRNAs Are Involved in Physiological Processes

Exosomal circRNAs not only participate in pathological processes but also play key roles in some physiological processes. For example, hsa_circ_0075932 has a low basal expression level in dermal keratinocytes, but its overexpression in these cells has no deleterious effect. However, if it is significantly expressed in adipose tissue, it directly combines with PUM2 to activate the AuroraA/NF-κB pathway, thereby promoting cell inflammation and apoptosis (135). In addition, Zhao et al. conducted experiments in mice, showing that exosomal circRNAs may participate in neuron growth and repair, nervous system development, and nerve signal transmission through glutamatergic synapses and cGMP-PKG signaling pathways (136). Therefore, it is necessary to further study exosomal circRNAs, which might help us better understand some important physiological processes.

## Summary and Prospect

Exosomes and circRNAs are both hot research topics. In this review, several important functions of circular RNA are summarized in detail. For example, circRNAs can act as miRNA sponges to affect gene expression, interact with Pol II to regulate the transcription of parental genes, act as a protein sponge to affect protein expression, and participate in protein translation through multiple pathways. Moreover, experiments have shown that circRNAs can enter exosomes. Although its regulatory mechanism is not completely clear, exosomal circRNAs play an important role in pathological processes such as tumors, nerves, cardiovascular diseases, as well as physiological processes such as inflammation and cell apoptosis. Exosomes are rich in lipid components such as cholesterol, neuramide, and sphingolipids, which are not easily degraded by proteases and ribonucleases, so circRNAs are stable in exosomes and can perform their functions. In addition, exosomes are found widely in various body fluids, such as blood, tears, saliva, sputum, semen, urine, and breast milk (137, 138). Therefore, liquid biopsies can be performed; it is convenient and simple for clinicians to take specimens and it is not traumatic for patients. Therefore, the study of exosomal circRNAs will provide a biological basis for the identification of new markers for early diagnosis, targeted therapy, and prognostic evaluation of various diseases in the future.

Even though significant breakthroughs have been made in the study of exosomes and circRNAs, there are still many unanswered questions. First, what conditions affect the interactions of circRNAs with other molecules, and what other types of modifications other than m^6^A in circRNAs are involved in protein translation? Second, are there differences between exosomes in cells and those in plasma, and do these differences affect the expression of circRNAs? Third, what is the specific mechanism by which circRNAs enter exosomes, and how are they cleared or involved in cell-to-cell communications? Fourth, blood, urine, sputum, and other liquids also contain many other impurities, including protein complexes and nucleic acid lysates, which interfere with the extraction of exosomes. Therefore, there are still many difficulties and challenges in the clinical application of exosomal circRNAs, and thus continuous in-depth research and exploration are needed.

## Author Contributions

JH and JL provided direction and guidance throughout the preparation of this manuscript. ZH, HC, and ZG wrote and edited the manuscript. JS and WL reviewed and made significant revisions to the manuscript. FX, YW, and SW collected and prepared the related papers. All authors contributed to the article and approved the submitted version.

## Funding

This work was supported by Science and Technology Project of Panyu District, Guangzhou (2019-Z04-02; 2020-Z04-026), Guangzhou Health and Family Planning Commission Program (No. 20181A011118; 20192A011027; 20191A011119; 20201A010085; 20212A010025), Guangzhou Science and Technology Plan Project (No. 202002030032), Medical Science and Technology Research Foundation of Guangdong Province (No. A2020304; A2022524), Scientific Research Fund project of Guangzhou Panyu Central Hospital (No. 2021Y002; 2021Y004).

## Conflict of Interest

The authors declare that the research was conducted in the absence of any commercial or financial relationships that could be construed as a potential conflict of interest.

## Publisher’s Note

All claims expressed in this article are solely those of the authors and do not necessarily represent those of their affiliated organizations, or those of the publisher, the editors and the reviewers. Any product that may be evaluated in this article, or claim that may be made by its manufacturer, is not guaranteed or endorsed by the publisher.
